# The Making of Corporate Legal Concession Theory^†^

**DOI:** 10.1093/ojls/gqad028

**Published:** 2024-01-10

**Authors:** Jonathan Hardman

**Keywords:** company law, separate legal personality, nature of the firm, concession theory, real entity, state gift

## Abstract

Professor Watson’s *The Making of the Modern Company* traces the development of the modern corporate form back to the East India Company, disproving a common notion that company law originated solely with small, private companies. This review article argues three key implications of this excellent book. First, Watson focuses on the duality of the modern company—with state-provided and private features. This cuts through, and goes a long way to resolving, the ongoing historic debate as to the nature of the company. Second, the primary unit of study chosen—the modern company—reminds corporate lawyers of our role in studying this duality in a very crowded field. Third, despite eschewing ‘concession theories’ of company law (which hold that the company is merely a concession from the state), Watson demonstrates a role for the state in the modern company that is often overlooked.

‘No amount of contracting can create a legal person.’^1^

## 1. Introduction

Company law scholarship often presents as a series of dichotomies. Competing narratives exist as to whether core constituents of the company are inherently ‘good’ or ‘bad’;^2^ whether companies should be run only in the interests of shareholders or serve a wider societal function;^3^ whether the company should be seen as public or private;^4^ and numerous other topics. One perennial^5^ and metaphysical^6^ topic is the company’s nature: is it merely the aggregate of its shareholders (aggregate theory), some autonomous real entity (real entity theory) or just something that arises as a result of concessions from the state (concession theories)?^7^ Professor Watson makes an important contribution to this debate in her excellent historically rooted book, *The Making of the Modern Company.* Watson’s approach removes some of the vagueness and metaphysicality by challenging core tenets in historical understanding of company law.

A UK narrative is that ‘company law has developed seamlessly from the law of partnership’.^8^ Under such narrative, the corporate form was never fully intended for one-person companies,^9^ but was instead voluntarily formed by small groups, with some modern problems arising due to scaling up the enterprise.^10^ Under this approach, the company conceptually springs from private ordering by shareholders. This connects to a particular strand of Anglo-American normative understanding of a company: that it represents a series of private, bilateral relationships,^11^ reducing the corporate form to a ‘nexus of contracts’,^12^ therefore trivial as it could be achieved by other methods.^13^ This approach inherently minimises the importance of a company’s separate legal personality, holding it as merely a ‘convenient heuristic formula’ for a bundle of rights,^14^ which at most lower the costs of private ordering.^15^ Normatively, then, company law should merely facilitate such private relationships,^16^ and its purpose is ‘responding to three principal sources of opportunism that are endemic to such organization’.^17^ There is a limited role for the state in this narrative—instead, company law is seen as responding to, and assisting where possible with, co-ordination hurdles between private parties.

This paradigm is not necessarily an accurate description of modern UK company law.^18^ Watson’s work demonstrates that it never has been—the ‘belief that the modern company started small based on its shareholders and then got bigger is widespread and not true’.^19^ Whilst parts of modern company law descend from partnership law, most parts appear from elsewhere. The first part of the book traces the historical development of the UK company and, by extension, UK company law. It argues that the modern company’s core features—separate legal personality, limited liability, and division between shareholders and directors (with the former having transferrable interests which receive a variable return and the latter tasked with managing the company) originated in the UK in the large, state-backed East India Company.^20^ Demonstrating this not only challenges the historical lineage of the contractarian school of thought in UK company law, but highlights the role that the state plays in the modern company. The state was core in creating the corporate form, so the modern company did not arise spontaneously out of private ordering^21^ utilising existing private law concepts.^22^ The second part of the book advances implications for our understanding of the modern company.

This review article makes three connected arguments in respect of Professor Watson’s book. First, it argues that the book makes an important contribution to the ancient debate as to the company’s nature by clarifying that it is multi-­faceted. Debates in this field tend to suffer from indeterminacy, where the same fact pattern can be extrapolated to a number of different positions^23^ and commentators attempt to present their approach as all-encompassing, missing important nuances within company law.^24^ Watson positions the company as having two aspects: its legal personality, which originates from the state; and its segregated corporate fund,^25^ which is private.^26^ The former encompasses legal rules in respect of the legal form, while the latter encompasses the general pooling of assets into a joint enterprise, which may or may not use the corporate form. This approach immediately cuts through the false dichotomies in the debate. Watson also connects this abstract debate to other debates in corporate law, particularly the need to conceptualise company law through property rights.^27^ This approach tethers the historical antecedent debate to more modern corporate law debates, re-emphasising its modern relevance. I argue, then, that in a crowded legal field as to the nature of the company,^28^ Watson has earned a place in the pantheon.

Second, law is not the only discipline exploring these concepts. The nature of a company is explored in (amongst others) economics, political science and sociology. This interdisciplinarity creates a conceptual challenge—should all disciplines take the same approach, or should different disciplines explore different ways of looking at the same or different aspects? Watson’s duality maps neatly onto the units of study of different disciplines. I argue that lawyers should focus on state provision of the separate legal person and related rules rather than the operation of the private fund, which is better studied by other disciplines. This is not to say, of course, that such state provision should not have a policy goal of furthering the private interest. However, Watson’s work acts as an important reminder about the key unit of study within company law, and how to differentiate corporate law study from non-legal disciplines. It also helps differentiate a corporate law understanding of legal personality from the approach adopted by other legal disciplines.

Third, I argue that the normative implications of the book, based on the foregoing, foreground the state in the future direction of company law research. Professor Watson has previously argued that the company’s separate legal personality arises as a concession from the state.^29^ By focusing on the duality that she identifies—balancing private interaction and a state-provided legal form and related rules—Watson backs away from this claim here. The normative implications, though, are that corporate lawyers should focus on the latter. This argument builds on the previous two—if the company has state-provided and private aspects, other disciplines study private aspects and the state-provided aspect is the key *legal* part, then company lawyers should focus on the state-provided aspects of company law. I have previously argued that concession approaches ‘became more of a straw man to be argued against, without many strong adherents’.^30^ Whilst Watson actively resists endorsing a concession view of the company,^31^ her approach lets us foreground the state, in turn increasing the state’s locus to intervene in corporate activity.^32^

These three arguments, taken together, mean that *The Making of the Modern Company* not only demonstrates how the company actually developed in the UK, but also sets the direction for a future company law research agenda: in oft-­misrepresented and maligned concession theories. Professor Watson thus does more than merely show where the company comes from. She creates the conceptual space for further exploration of the state’s role in fundamental company law.

The rest of this review article proceeds as follows. Section 2 outlines the duality identified by Watson as to the nature of the company, and its importance. Section 3 argues that Watson’s approach outlines the role that law should play in an interdisciplinary area. Section 4 explores how these parts build to create normative implications for a future direction of company law research. Section 5 concludes.

## 2. A Place in the Pantheon

‘The combination of a legal form derived from the state with a private fund seeded by money provided by investors anticipated the modern company.’^33^

The book has two parts. The first focuses on the doctrinal and practical development of UK business forms.^34^ Roman law had two key business forms: a small form, available by agreement; and a large form, available for state interactions.^35^ Watson has previously argued that English law followed; that the modern company is a hybrid between the two types of vehicle; and that modern company law is also such a hybrid.^36^ Here, she unpacks that hybrid. She identifies the two aspects of such hybrid: the artificial legal person, provided by the state; and the corporate fund held in it, the private element that produces a profitable return for those who invest in it.^37^ Without the state-provided artificial legal person, there would be no modern company.

The state-provided form alone was not enough on its own to create the modern company—it needed to be coupled with the corporate fund. The state-­provided entity is the shell which is utilised by the private fund. This shell has a myriad of legal rules attaching to it—such as limited liability for shareholders,^38^ and rules in respect of how the shell can issue shares and raise money.^39^ The private fund, though, is what uses this shell. It arises whenever individuals decide to jointly dedicate resources towards a common goal, and became more sophisticated when accounting rules facilitated the segregation of such joint assets from individual assets.^40^ Watson shows that the state-established East India Company was the first English company to have permanent capital,^41^ allowing the efficient segregation of the corporate fund from individual and fluctuating shareholders. This was facilitated by the creation of double-entry bookkeeping,^42^ the ‘architecture that isolates the Corporate Fund’.^43^ This fund need not use the corporate legal form—it could easily use any one of a number of alternative vehicles, for example partnership forms^44^ or trust arrangements.^45^ Without the fund using the state-provided corporate form, though, it would not be the primary vehicle for business. The creation of today’s company, then, relied upon private innovation being coupled with provision of a state-backed permanent entity.

This takes us to the nature of the company. Trying to distil the company down to its core essence^46^ was a ‘virtual obsession’ in the United States for 50 years, until it declined towards the end of the 1920s.^47^ There are a number of different sub-debates,^48^ but at its core is whether the company is merely the aggregate of its shareholders (aggregate theory),^49^ is something that merely arises as a concession from the state (concession theory)^50^ or is something real and autonomous from its shareholders and the state (real entity theory).^51^ The debate started in the context of German unification in the second half of the nineteenth century.^52^ In the UK, Maitland and Pollock stridently advanced a real entity approach.^53^ There were two key problems with argumentation structures within the historic debate. First, commentators were cherry-picking both facts and normative implications of those facts to achieve their desired ends—meaning that the same features could be used to reach opposed conclusions.^54^ Second, positions tended to be less clear cut than presented by most commentators—meaning that reality operated somewhere in the middle of the extreme positions taken.^55^

Yet the debate was—and remains—important. Law’s understanding of the company is important for the internal consistency (and connected legal certainty) of both company law and the wider legal system.^56^ This is especially so in the area of legal personality, where company law must connect with other areas of the legal taxonomy, such as criminal law.^57^ Watson’s analysis means that the company is not neatly described by any one approach taken in the historical debate. Instead, it has a duality between the state-provided legal form and the private fund which utilises it. Other commentators have previously discussed a duality in a company, between the business part of the company and the state-provided legal shell through which it operates.^58^ Watson proves this and operationalises it. As most commentators in the field present a single approach as the superior way to conceptualise the company,^59^ demonstrating this duality is a major methodological development for the field.

The state-provided aspect of the modern company is frequently ignored.^60^ This is no doubt because the company is seen as merely a method by which private ordering is achieved.^61^ This approach holds that the method is secondary to the fundamental study of such ordering. More than this, there is an assumption that most jurisdictions provide companies with the same legal characteristics. The logic is that as shareholders choose a company’s country of incorporation,^62^ jurisdictions will need to appeal to shareholders in order to attract incorporations.^63^ The result of market dynamics between jurisdictions is argued to be the homogeneity of corporate laws, with a shareholder-friendly bent.^64^ As companies are selected from a menu of business vehicles, should the corporate form cease to be beneficial for business, business would transfer to alternative vehicles and the economy would continue almost unchanged,^65^ as would the future direction of economic activity.^66^ Under this approach, company law becomes just a function of investor interests and so inherently less interesting—merely trivial, setting the rules for one method to achieve private ordering.^67^

Watson, though, identifies that this whole cycle started with the state. All features that we identify in a modern company were originally inherent in the English East India Company,^68^ a state-chartered corporation.^69^ Such legal forms originally required some form of ostensible public purpose,^70^ and were not possible without express state approval.^71^ This means that, rather than starting small and scaling up, UK companies started big, with smaller entities mimicking their features.^72^ Smaller, private-originated deed-of-settlement companies attempted to copy the corporate form and its state-granted features.^73^ This means that the modern company is, ultimately, descended not from small companies, but from the large, state-provided corporate form. It also means that at the heart of understanding modern private ordering lies the state: the state provided something that actively facilitated private ordering, rather than existing private ordering utilising the company form.^74^

This theme is developed by Watson. Early business entities relying purely on private ordering—such as deed-of-settlement companies—could not achieve all the features of the corporate form.^75^ Modern defendants of company law as a non-trivial academic subject tend to defend it on the grounds that it separates assets of, and claims against, investors from assets of, and claims against, the company.^76^ This is said to be predicated upon property rules rather than contract.^77^ The implication is thus that company law is merely a combination of parts of the ‘primary’ private law taxonomy—a combination of property and contract rules.^78^ Whilst this approach removes the triviality of corporate law, it still undermines the autonomy of company law—which under this approach remains conceptually subject to these other private law rules.^79^

Watson demonstrates that company law cannot perform its magic without state provision of a separate legal entity.^80^ The modern company started with the state, and still obtains its core characteristics from the state. Watson identifies that the modern company is not merely an amalgam of pre-existing private law features stumbled upon by private ordering. Instead, these features are bestowed by state action.^81^ This challenges the idea of autonomous private ordering being ambivalent as to the organisational forms presented to it—the state was required to create, and provide, a form of vehicle with the features of the modern company.

Starting with the state does not mean ending with the state, though. It is the corporate fund that provides something real. The state provides the shell, into which real things can be poured. Not all shells will have real things in them.^82^ However, once it has a real thing in it, it then has real effect in society. As a result, for Watson, the state aspect does not encapsulate the entirety of corporate life. It reflects the origin of the modern company,^83^ but not all corporate activity. Synthesising the initial state provision of the corporate form with ongoing private activity avoids the false dichotomies of historical debates: the company initiated from the state, yet operationalises through private action. Without private action, the company would never be used; without state provision of legal form, the private fund would need to operationalise through other means.

There is thus truth in arguments that the company can be something real, and that it is linked to the state. This helps resolve the historic debate whilst also connecting to more modern debates as to the role of company law. For this reason, *The Making of the Modern Company* and its author should be admitted to the pantheon of commentators in a crowded field. In proving the concept that the company has two sides, and unpacking the relationship between the two, Watson’s work helps resolve key ambiguities in the debate over the nature of the company. A modern company is an artificial legal person, originating from the state, which facilitates the lock-in of a private corporate fund that can grow perpetually. It takes the hunch that there are multiple facets to the company^84^ and operationalises it. It allows a clear, universal conception of the nature of the company without sacrificing nuance. Is an operating company a real entity or does its form derive from the state? Yes to both. Orts argues that the two approaches reflect ‘bottom up’ and ‘top down’ approaches to the company respectively.^85^ Watson demonstrates that both are present in modern companies that operate in the world, cutting through the debate.

## 3. A Very Legal & Corporate Analysis

‘The risk with ignoring the law is that the significance of key legal characteristics is not sufficiently recognised.’^86^

The second development that *The Making of the Modern Company* provides relates to the role of corporate law in exploring these debates. The challenge for corporate law is making a *unique* contribution on the nature of the company, a field studied by many. There are two fronts on which corporate law needs to delineate itself—from non-legal academic disciplines and from other parts of the legal taxonomy. Failure to delineate on the first ground leads to missing the importance of legal features of the corporate form, and failure to delineate on the second leads to missing the corporateness of analysis. Watson’s approach clarifies these faultlines. It lets corporate lawyers examine the state-provided aspect of personality and related rules, whilst also retaining the distinct corporate aspects of the corporate fund.

First, many non-legal disciplines explore the nature of the company.^87^ All do so from within their own disciplines. Watson focuses on the primary corporate legal business form in the UK, being the company.^88^ A company has a technical meaning in the UK, being a specific vehicle created under a specific piece of legislation.^89^ The company is thus a UK company lawyer’s main unit of study, and is clearly defined. This is not the case for other disciplines. Economists focus on a different concept—the firm.^90^ The precise extent and definition of the firm is rather vague.^91^ Watson’s duality opens the possibility that different disciplines could be looking at different facets of corporate life, and ultimately different units of study.^92^

Through Watson’s lens, it is evident that the artificial legal person can exist without a meaningful corresponding corporate fund (ie without being part of a firm), and conversely that segregated funds for investment can exist without artificial legal persons (ie firms through legal forms that are not companies^93^). The former are companies, and are thus subject to company law and should be studied under it.^94^ The latter are not companies, and thus are not subject to company law. Company lawyers, then, need to cater for the archetypal entity, in which both are present, and also the entity which does not undertake any meaningful activity, so could be said to have no attached firm. The only subject flowing through all subjects of company law is the provision of an artificial legal person by the state—it is the ‘lowest common denominator applicable to all companies’^95^—and its associated rules. These rules facilitate the creation of the corporate fund, but do not demand it.^96^ Axiomatically, the unit of study for economists is the firm. They therefore care not (or little) about empty companies, but do care about firms not using the corporate form. Watson’s duality, then, indicates that different disciplines are likely to focus on different aspects of this duality. In particular, lawyers seem best placed to focus on the state-provided aspect of the artificial legal person, which facilitates—but does not constitute—economic activity.

These two aspects remain linked. Deakin and others argued that firms are ‘creatures of law’.^97^ They went on to argue that the ‘glue’ of the firm is the law that creates a separate legal person.^98^ If your unit of study is the firm (ie the deployment of private funds in joint enterprise) and you focus on those utilising the corporate form rather than, say, a partnership form,^99^ then this is correct. In other words, if you are only looking at real things operating in the world through companies, then this approach borders tautology. This is not, though, the position for all companies, so not a universal position for company law. Watson’s insights let lawyers conceptualise the creation of an artificial person first, which then *can* operate in the world based on a corporate fund utilising it.^100^ This once again helps focus the corporate legal approach on the unit of study. Corporate lawyers should look to the provision of the artificial legal person.

Watson’s approach lets us avoid a false syllogism for company lawyers trying to engage with this body of literature. There is a risk that lawyers following the language of economists conclude that not only is every firm a company, but *every company is a firm*. Economists do not mean this,^101^ and yet make statements to that effect.^102^ Companies and firms are not entirely unrelated concepts—so the firm and the company do not exist in separate universes.^103^ Watson demonstrates that such legal tools as permanent personality^104^ and permanent capital^105^ allowed the lock-in of funds for the long term. Thus, Watson’s argument is that corporate law facilitated the success of firms, connecting to the approach of Deakin and others. As such, the firm does not merely use the corporate form^106^—the firm itself is shaped by the company, and thus by state-provided company law.

Watson’s duality is helpful for focusing the unit of study for different disciplines. Without a clear understanding of the units of study that we are focusing on, there is a risk of confusion between audiences. For example, when Jensen and Meckling created the ‘nexus of contracts’ concept,^107^ they applied it, at various times, to the company and/or the firm.^108^ This creates an ambiguity of meaning as to which, exactly, they were arguing operated as a nexus. It seems most likely that they were envisioning a situation whereby the boundaries of the two were the same.^109^ Yet this lack of clarity over exactly what they were focusing on risks corporate lawyers misunderstanding their insights.^110^ Ambiguities abound—business scholar Mayer has advanced arguments as to the role of company law^111^ that lawyers have argued misunderstand the field.^112^ This is a risk for work between law and economics, where overlapping terminology often discusses different underlying concepts.^113^ Our challenge, then, is to ensure, when looking at an area of crossover, that we do so with our own specialism in mind, and are cognisant of what other disciplines are studying and why.^114^ Political science also debates the role of law and the state in the company.^115^ Thus, economics and political science are increasingly emphasising law’s role in their fields. It is therefore important that corporate lawyers follow Watson’s example by providing clear analysis of the legal provision of law’s unit of study—the artificial legal person—for these fields to utilise.

By focusing overtly on the unit of study—the company—Professor Watson focuses corporate legal analysis on the company. In particular, Watson focuses on the legal rules provided by company law that are not available through purely private ordering—legal benefits that can *only* be obtained by using the artificial legal person and not by other legal methods for the private fund to operationalise through.^116^ The natural implication of Watson’s duality, then, is that different parts of that duality should be explored through different disciplines, with the provision by the state of the artificial legal person and associated rules falling neatly within law’s purview. This analysis can, of course, use normative insights from those disciplines which study the corporate fund.^117^ However, to be helpful to other disciplines which are increasingly reaching towards law for answers to questions within their own subjects, it is important that company law understands its unit of study clearly. Watson’s analysis provides a method to do so: legal rules—provided by the state—create a legal framework under which economic activity can operate (a private fund). Company lawyers have evident expertise to look at the former, and those studying business activity have evident expertise to look at the operation of the latter. Of course, both speak to each other (the form influences the activity, and the nature of the activity needs to influence developments of the form), and are part of the holistic understanding of the company, but they remain conceptually separate. To ensure a legal analysis, then, Watson’s duality implies that company lawyers are best situated to focus on the creation of the artificial legal person.

The second challenge for company law is to identify how company law’s legal analysis is delineated from other parts of the legal taxonomy which look at legal personhood.^118^ It is tempting for corporate lawyers to defer to this body of literature on law’s persons generally, where corporate law’s understanding often jars with broader study.^119^ More than this, to focus on only the artificial legal person as part of law’s spectrum of persons^120^ relegates the study of company law to secondary application of other legal disciplines. There is something instinctively special and distinctive about the corporate legal person, though. It has long been advocated that other bodies, such as sports clubs, should obtain the same legal personality as the company.^121^ Yet attempts to do so have mixed results, because any attempt to do so needs its own normative foundation.^122^ Watson provides such a foundation for the company—the company is not *just* an artificial legal person, but instead one that facilitates a perpetual corporate fund. Thus, whilst company lawyers should study the company’s personality, they should do so with an eye to the provision of that fund, which other legal areas do not need to concern themselves with.

Part of the company’s legal personality is as a home for the corporate fund.^123^ This differentiates the company’s legal personality from other classes of legal personality. This approach will help add clarity to questions such as whether companies should have criminal liability,^124^ or human rights.^125^ However, it reduces the need for the corporate legal person to fully follow other understandings of legal personhood.^126^ Watson separates the concepts of the company as a juridical person (which must cohere with general legal personhood understanding) from it being a separate legal person from its shareholders and investors (a unique issue for company law).^127^ They are, as Watson indicates, ‘two related, but different, concepts’.^128^ Treating them as such means that there is something uniquely corporate about the legal analysis of the company’s separate legal personality. Watson demonstrates that these two concepts exist within the modern company, so any study of modern company law must include both. So, in addition to helping lawyers ensure that their understanding of the company is legal, Watson’s duality allows corporate lawyers to ensure that their discussion of legal personality is distinctly corporate. It therefore is different from general considerations of law’s persons. Watson’s is not the only roadmap to achieve this,^129^ but it does follow neatly from the duality she identifies in the nature of the company. It therefore sows the seeds to set up normative implications of the book for the future development of company law analysis.

## 4. A Rejected Normative Implication

‘Invisible, immortal beings are amongst us, determining and controlling many aspects of our lives.’^130^

So far, then, I have argued that Professor Watson’s work is important because it acknowledges a duality in the nature of the company—part state-created, part private—and that companies cannot exist without the former but can without the latter. In an interdisciplinary field, it is more natural for company lawyers to focus on state provision of legal personality and associated legal rules. This section argues that the normative implications *for law* of *The Making of the Modern Company* are that it should examine state-provided aspects of the modern company—company law normatively follows a concession theory, even if other disciplines look to other theories to explain their units of study. After all, the legal aspect of Watson’s duality—the provision of artificial legal personality—is the part connected to the state.

Professor Watson eschews concession theories on the grounds that general incorporation statutes^131^ make incorporation ‘now a right, not a concession’.^132^ Watson has previously convincingly argued that general incorporation changed the *process* by which companies were incorporated, but not the fact that incorporation is provided by the state.^133^ In *The Making of the Modern Company*, this argument is not fully followed—instead, we are told that incorporation starts by the private acts of shareholders, through a state process, which creates a legal person, which can then become real.^134^ Watson may be right that concession theories do not fully explain the nature of the company, following her dual nature of the company. Yet, by deploying her duality, and factoring in different focuses of study as per the previous part, we can identify that the legal parts of Watson’s duality are best explained by concession theories. Thus, for company law, concession may be the best-fitting paradigm.

Watson persuasively argues that the features of the modern company could not arise without the state providing company law.^135^ To the extent that shareholders are able to establish companies, and that companies can operate to maximise the private fund, it is because company law—set by the state—allows it. As such, if company law should focus on the part of Watson’s duality that relates to the state creation of an artificial legal person, then it should inherently focus on the concessions given to facilitate the private fund by company law. As a matter of UK company law, the ‘mysterious rite’ of incorporation^136^ involves two steps. First, the Registrar of Companies reviews documents submitted to them against statutory requirements and, if satisfied, registers them.^137^ Second, upon that registration, a certificate is issued stating that the company exists, which creates the company.^138^ Whilst the second happens automatically following the first, the first is a check by the state, processed by the state. There is no right to obtain a corporate form should such checks not occur, or if the paperwork is lost. These checks are about to become more stringent.^139^ Both the process for incorporation and its operation rely on state actors.

As such, we have a situation whereby there are two core parts to the modern company: the artificial legal person and the corporate fund that can exist within it. The former comes from the state, and the latter is affected by the former. All companies require the former—without the former, it is not a company—whereas the latter is contingent upon the company operating in the world.^140^ Law’s natural focus is on the former—the artificial legal person, provided by the state, and the legal rules applicable to it. This corporate form is often argued to be a considerable force for harm in society, by deploying collectivised power to the detriment of others.^141^ Part of studying this artificial legal person must be working out how to resolve its harms. Watson states:

States may not require corporations to compensate for the externalities they create. The law may not even recognise that the essential characteristics that make the corporate form so potent, in particular status as a legal person, are ultimately derived from the state. This matters, because it relates to the rights of states in respect to modern companies. It provides a rationale for state regulation of corporations.^142^

This is very difficult to argue with. It leads to a slight tension, though. Watson makes the case that the legal features of the modern company originated in a state-backed entity, has previously argued that the artificial legal person on which it is all predicated comes from the state and in this quote decries that law may not ‘recognise’ the state’s role (implying the state has such a role). Yet, she does not fully adopt the theoretical school that flows from here—that the legal features of the modern company are best conceptualised as concessions from the state. Watson refers to companies in a number of places as being ‘endowed entities’,^143^ but, by rejecting concession theories, is at times unwilling to embrace the state-centric legal implications that flow from such endowments coming from the state. The difference appears mostly semantic—whether it is labelled as a concession theory or not, the state’s role in corporate life is foregrounded by Watson’s analysis.^144^

Yet, expressly embracing concession theories for company law would change the normative conclusions drawn by Watson from the analysis. The diagnosis remains the same—companies can cause great harm in the world, through the aggregation and deployment of power of corporate funds (facilitated by state action through company law).^145^ Watson states:

Until recently, states have seemed powerless to regulate or control [big companies]. The veil must fall from our eyes … Corporations have been described as Frankenstein’s monsters, and in many ways they are artificial persons created by Man. Just as we make them, we can unmake them or remake them in a form that serves us all rather than the gilded few, as we engage in the grand challenges the world confronts.^146^

Focusing on the legal concessions by the state to the company’s artificial legal person to facilitate private wealth through the corporate fund would answer this call to arms. Denying the force of concession theories naturally limits and weakens the tools to deal with this. One of Watson’s proposals is to focus on the artificial legal person’s perpetuity—freeing boards to act sustainably in the long term.^147^ These, of course, are the same boards who are provided a large degree of deference in the Anglo-American world anyway,^148^ and who have either contributed to or at least not solved current issues.

A stronger normative position would have been to embrace the notion of this artificial person being, and consisting of, legal concessions to business from the state, as in Watson’s previous work. This retains Watson’s duality—it merely means that company law should focus on the concessions given by law, whatever conceptual approaches other disciplines adopt. The state provides the corporate form either by direct provision or by providing the framework through which incorporation occurs, and sets the rules under which it operates (via company law for internal matters). In either event, the state is able to change the process and the requirements for such incorporation. Under either approach, the provision and regulation of the artificial legal person upon which the modern company is predicated—and which shapes the corporate fund that sits within it—is from the state. Either approach provides the space for the state to make one of two adjustments in respect of the terms of its legal concession.

First, it could restrict the circumstances in which such concession is granted. This could occur in many ways. It could occur as an incorporation requirement, or at any stage of corporate life. The state could intervene where it sees harm being caused by the artificial legal person, however small, by restricting it. It could thus act as an ultimate sanction for a company abusing its gifted features in ways that fall short of outright illegality. After all, the private aspect of the corporate fund generates a considerable amount of profit for the insiders to the fund. It is able to do so due to features endowed upon it by the state. The state therefore must have the locus to remove or deny such features where harm falling short of illegality outweighs good. Other conceptual approaches which do not explore concessions would require a higher hurdle for the state to intervene in what is perceived as something real and autonomous, or an aggregation of private law rights.

Concession approaches allow state intervention to limit any concession associated with separate legal personality—for example, by limiting aspects of shareholder limited liability in certain circumstances.^149^ It could limit the activities of companies. This sounds radical, but actually reflects reality: the state currently gatekeeps what a company can and cannot do. We consider it self-evident that a company cannot marry or have children. A company cannot itself be a medical doctor.^150^ These examples demonstrate that the state already limits the extent of its legal concession of the artificial legal person. However, without a concessional approach, such limitations are not fully conceptualised. *The Making of the Modern Company* lays considerable groundwork in doing so, even if its author does not fully embrace this conceptualisation.

Second, the state could extract counter-concessions from the corporate fund for the privileges endowed to it by the deployment of an artificial legal person. These counter-concessions already exist on a number of levels. UK law requires all limited companies to include the word ‘limited’ in their name.^151^ Rules restrict withdrawals from the corporate fund.^152^ Most importantly, we tax the artificial legal person on the corporate fund’s profits, then also tax shareholders on the dividends they receive from it.^153^ This is key evidence of the state requiring something back (a counter-concession) in exchange for providing the perpetual artificial legal person to hold the corporate fund. Viewing it as such helps to strengthen the state’s hand to regulate the company through company law—it already heavily does so. The presence of such counter-concessions opens the space to adjust such counter-concessions, and demand more where required.

Concession theories do not purely demand restrictions on the operation of companies. The state grants the concession of companies because their activities benefit society.^154^ It is therefore entirely legitimate under this argumentation structure to argue for further proliferation of greater concessions to achieve societal benefits.^155^ These concessions, though, facilitate the concentration of power and private wealth,^156^ and encourage harm to third parties.^157^ The purpose of the particular legal concession theory outlined here is that we need to constantly balance the benefits obtained from state provision of the corporate form and associated legal concessions *against* the harms caused—holistically and on an entity-by-entity basis.^158^ We may therefore have greater limitations/require greater counter-concessions from those causing greater harms. So long as the societal benefits outweigh the societal harms, the operation of the company should be unaffected, or should obtain even greater state concessions. However, once that benefit is outweighed, the state has the locus to intervene. Indeed, under such conceptualisation, it has the *duty* to intervene, as the combination of features that cause such harm are ultimately endowed by the state.

This is heavily implicit in *The Making of the Modern Company*. Even though Professor Watson does not overtly follow a concession theory, this is the logical conclusion of a number of parts of the analysis advanced. The state provides an artificial legal form for a private fund to operationalise in. Private funds can use any number of alternative paths to achieve their ends. This could be used as a reason to not change corporate law, as that risks a flight to alternative vehicles.^159^ Implicit in such an argument, though, is that there is something advantageous about the company form. That form originates from state activity. In other words, the argument evidences that the state currently provides a myriad of features which enable private advantage. The first recognisable modern company, Watson proves, was the state-created East India Company. Since that company was formed, private funds have attempted to emulate those features. However, they were not able to do so without state action. State action is still required to incorporate a company. It is a state actor who undertakes the incorporation, in accordance with a process laid down by the state which can be altered (in form or substance) by the state.

To achieve Watson’s end goals—reining in the excesses of egregious companies without losing their advantages; remaking them in a form that suits the needs of society—a concession theory is the answer. Factoring the previous part’s interdisciplinary part into the analysis makes it clear that it is entirely coherent to hold that whilst there is an inherent duality in a modern company, it is the role for lawyers to look at one part of that duality, from a particular conceptual viewpoint. Thus, from a perspective of historical antecedent, and theoretical argumentation, Professor Watson is laying the ground for future corporate *legal* concession theories, even if other disciplines embrace real entity or aggregate approaches.

## 5. Conclusion

*The Making of the Modern Company* makes a number of key advances to company law theory. First, it highlights, conceptualises and operationalises a duality at the heart of the modern company. It is an artificial legal person, whose features (including its perpetuity) facilitate and further a corporate fund. This goes a long way to resolving historical and ongoing theoretical debates as to the nature of the company, and connects them to corporate reality. As such, it will be a major text for those engaging with this theoretical debate. This duality has an interdisciplinary effect; lawyers should study one part of it. This navigates between the Scylla of company law losing its legal focus and the Charybdis of company law losing its corporate focus within law. It thus provides methodological insights which can be followed to strengthen the autonomy of company law against all comers. It is a must-read book.

It shies away, though, from an evident extrapolation of its analysis to a normative framework for company law that resolves its highlighted problem. This analysis, based on Professor Watson’s previous work, is easy to complete. Even though a concession theory is not the key takeaway intended for the book, it sows the seeds for a particular legal concession theory, and future writers in all disciplines from that theoretical school will draw from it. As such, however inadvertently it may be, *The Making of the Modern Company* is also the making of a corporate legal concession theory.

## Footnotes

1 *MMC* 221.

2 See JG Hill, ‘Good Activist/Bad Activist: The Rise of International Stewardship Codes’ (2018) 41 Seattle UL Rev 497; J Hardman, ‘Succession’s Lessons for the UK’s Dual Class Shares Debate: Beyond the Founder as the Benign Genius’ (2022) Law, Culture and the Humanities <https://doi.org/10.1177/17438721221080784> accessed 7 August 2023.

3 Compare CP Mayer, *Prosperity: Better Business Makes the Greater Good* (OUP 2018) with LA Bebchuk and R Tallarita, ‘The Illusory Promise of Stakeholder Governance’ (2020) 106 Cornell L Rev 91.

4 eg A Wolfe, ‘The Modern Corporation: Private Agent or Public Actor?’ (1993) 50 Washington & Lee Law Review 1673; WT Allen, ‘Our Schizophrenic Conception of the Business Corporation’ (1992) 14 Cardozo L Rev 261.

5 R Harris, ‘The Transplantation of the Legal Discourse on Corporate Legal Personality Theories: From German Codification to British Political Pluralism and American Big Business’ (2006) 63 Washington & Lee Law Review 1421; MH Hager, ‘Bodies Politic: The Progressive History of Organizational “Real Entity” Theory’ (1989) 50 U Pitt L Rev 575.

6 BR Cheffins, *The Trajectory of (Corporate Law) Scholarship* (CUP 2004) 38–40.

7 GA Mark, ‘The Personification of the Business Corporation in American Law’ (1987) 54 U Chi L Rev 1441; D Gindis, ‘From Fictions and Aggregates to Real Entities in the Theory of the Firm’ (2009) 5 Journal of Institutional Economics 25.

8 *O’Neill v Phillips* [1999] 1 WLR 1092, 1099A. See *MMC* 152.

9 Conceptual issues remain—S Worthington, ‘Corporate Attribution and Agency: Back to Basics’ (2017) 133 LQR 118.

10 eg separation of ownership and control—see AA Berle and G Means, *The Modern Corporation and Private Property* (rev edn, Harcourt, Brace & World 1967) chs 1 and 2.

11 M Klausner, ‘The Contractarian Theory of Corporate Law: A Generation Later’ (2006) 31 J Corp L 779.

12 M Jensen and W Meckling, ‘Theory of the Firm: Managerial Behaviour, Agency Costs and Ownership Structure’ (1976) 3 Journal of Financial Economics 305; D Gindis, ‘On the Origins, Meaning and Influence of Jensen and Meckling’s Definition of the Firm’ (2020) 74 Oxford Economics Papers 966.

13 BS Black, ‘Is Corporate Law Trivial?: A Political and Economic Analysis’ (1990) 84 Northwestern University Law Review 542.

14 R Kraakman and others, *The Anatomy of Corporate Law: A Comparative and Functional Approach* (3rd edn, OUP 2017) 2.

15 OE Williamson, ‘The Economics of Organization: The Transaction Cost Approach’ (1981) 87 American Journal of Sociology 548.

16 F Easterbrook and DRR Fischel, ‘The Corporate Contract’ (1989) 89 Colum L Rev 1416.

17 Kraakman and others (n 14) 2.

18 eg D Gibbs-Kneller, D Gindis and D Whayman, ‘“Not by Contract Alone”: The Contractarian Theory and the Paradox of Implied Terms’ (2022) 23 EBOR 573; MT Moore, ‘Private Ordering and Public Policy: The Paradoxical Foundations of Corporate Contractarianism’ (2014) 34 OJLS 693.

19 *MMC* 1.

20 *MMC* ch 5; N Robins, *The Corporation That Changed the World: How the East India Company Shaped the Modern Multinational* (Pluto 2012).

21 On the company as being merely a facet of private ordering, see OE Williamson, ‘Assessing Contract’ (1985) 1 J L Econ & Org 177.

22 J Morley, ‘The Common Law Corporation: The Power of the Trust in Anglo-American Business History’ (2017) 116 Colum L Rev 2145.

23 See J Dewey, ‘The Historic Background of Corporate Legal Personality’ (1926) 35 Yale LJ 655.

24 A Nékám, *The Personality Conception of the Legal Entity* (Harvard UP 1938) 28–33.

25 On which, see SM Watson, ‘The Corporate Legal Entity as a Fund’ [2018] JBL 467.

26 *MMC* 4–9.

27 J Armour and MJ Whincop, ‘The Proprietary Foundations of Corporate Law’ (2007) 27 OJLS 429.

28 A moratorium has been proposed on the subject—see B Bouckaert, ‘Corporate Personality: Myth, Fiction or Reality?’ (1991) 25 Israel Law Review 156, 156.

29 SM Watson, ‘The Corporate Legal Person’ (2019) 19 JCLS 137.

30 J Hardman, ‘Fixing the Misalignment of the Concession of Corporate Legal Personality’ (2023) 43 LS 443, 448.

31 *MMC* 212–15.

32 J Hardman, ‘Looking Beyond Separate Legal Personality, or How Many Titles have Rangers Won?’ [2022] Jur Rev 1, 4–8.

33 *MMC* 26.

34 *MMC* 15–155.

35 H Hansmann, R Kraakman and R Squire, ‘Law and the Rise of the Firm’ (2006) 119 Harv L Rev 1333.

36 SM Watson, ‘How the Company Became an Entity: A New Understanding of Corporate Law’ [2015] JBL 120.

37 *MMC* 7.

38 Insolvency Act 1986, s 74.

39 See L Gullifer and J Payne, *Corporate Finance Law: Principles and Policy* (3rd edn, Hart Publishing 2020) ch 10.

40 WL Raby, ‘The Two Faces of Accounting’ (1959) 34 The Accounting Review 452.

41 *MMC* ch 3.

42 RA Bryer, ‘The History of Accounting and the Transition to Capitalism in England: Part 2 Evidence’ (2000) 25 Accounting, Organizations and Society 327. See discussion in *MMC* 144–6.

43 *MMC* 9.

44 See L Macgregor, ‘Partnerships and Legal Personality: Cautionary Tales from Scotland’ (2020) 20 JCLS 237.

45 EC Chaffee, ‘Justifying Business Trusts’ (2023) 64 BCL Rev 523.

46 eg G Teubner, ‘Enterprise Corporatism: New Industrial Policy and the “Essence” of the Legal Person’ (1988) 36 Am J Comp L 130; K Cowling and R Sugden, ‘The Essence of the Modern Corporation: Markets, Strategic Decision-Making and the Theory of the Firm’ (1998) 66 The Manchester School 59.

47 MJ Horwitz, *The Transformation of American Law, 1870–1960: The Crisis of Legal Orthodoxy* (OUP 1992) 101.

48 At least 16 schools of thought can be identified—M Wolff, ‘On the Nature of Legal Persons’ (1938) 54 LQR 494, 496.

49 eg V Morawetz, *A Treatise on the Law of Private Corporations Other than Charitable* (Little, Brown & Co 1882).

50 For modern discussion, see SJ Padfield, ‘Rehabilitating Concession Theory’ (2014) 66 Oklahoma Law Review 327.

51 See E Micheler, *Company Law: A Real Entity Theory* (OUP 2021).

52 Harris (n 5).

53 FW Maitland, ‘Moral Personality and Legal Personality’ (1905) 6 Journal of the Society of Comparative Legislation 192; F Pollock ‘Has the Common Law Received the Fiction Theory of Corporations?’ (1911) 27 LQR 219.

54 Dewey (n 23).

55 M Radin, ‘The Endless Problem of Corporate Personality’ (1932) 32 Colum L Rev 643.

56 S Deakin, ‘Juridical Ontology and the Theory of the Firm’ in F Gagliardi and D Gindis (eds), *Institutions and Evolution of Capitalism* (Edward Elgar 2019).

57 eg CMV Clarkson, ‘Kicking Corporate Bodies and Damning Their Souls’ (1996) 59 MLR 557.

58 P Vinogradoff, ‘Juridical Persons’ (1924) 24 Colum L Rev 594. See discussion in Hardman, ‘Fixing the Misalignment’ (n 30).

59 RJG Claassens, ‘Political Theories of the Business Corporation’ (2022) 18 Philosophy Compass e12892.

60 *MMC* 8.

61 OE Williamson, ‘Comparative Economic Organization: the Analysis of Discrete Structural Alternatives’ (1991) 36 Administrative Science Quarterly 269.

62 R Romano, ‘Law as a Product: Some Pieces of the Incorporation Puzzle’ (1985) 1 J L Econ & Org 225.

63 O Bar-Gill, M Barziza and L Bebchuk, ‘The Market for Corporate Law’ (2006) 162 Journal of Institutional and Theoretical Economics 134.

64 H Hansmann and R Kraakman, ‘The End of History for Corporate Law’ (2001) 89 Geo LJ 439.

65 AA Berle, *The 20th Century Capitalist Revolution* (Harcourt, Brace 1954) 31–7; WW Bratton, ‘The “Nexus of Contracts” Corporation: A Critical Appraisal’ (1989) 74 Cornell L Rev 407.

66 See P Ireland, ‘Capitalism without the Capitalist: The Joint Stock Company Share and the Modern Doctrine of Separate Corporate Personality’ (1996) 17 Journal of Legal History 41.

67 Black (n 13).

68 *MMC* ch 5.

69 See PJ Stern, ‘The English East India Company and the Modern Corporation: Legacies, Lessons, and Limitations’ (2016) 39 Seattle UL Rev 424.

70 Although this was often abused—see M Patterson and D Reiffen, ‘The Effect of the Bubble Act on the Market for Joint Stock Shares’ (1990) 50 Journal of Economic History 163.

71 See HN Butler, ‘General Incorporation in Nineteenth Century England: Interaction of Common Law and Legislative Processes’ (1986) 6 International Review of Law and Economics 169, 171–80.

72 For parallel dynamics later, see R Harris, ‘The Private Origins of the Private Company: Britain 1862–1907’ (2013) 33 OJLS 339.

73 For the precise legal features combined in such ‘companies’, see the discussion in A Televantos, *Capitalism Before Corporations: The Morality of Business Associations and the Roots of Commercial Equity and Law* (OUP 2020).

74 *MMC* 44–5 discusses the importance of political context in the success of the East India Company.

75 *MMC* 125–6.

76 See H Hansmann and R Kraakman, ‘The Essential Role of Organizational Law’ (2000) 110 Yale LJ 387; MM Blair, ‘Locking in Capital: What Corporate Law Achieved for Business Organizers in the Nineteenth Century’ [2003] UCLA Law Review 387.

77 Armour and Whincop (n 27).

78 Working in tandem with trusts, Maitland’s ‘blessed back stair’ of the law—O Gierke, *Political Theories of the Middle Age* (FW Maitland tr, CUP 1913) xxxi. See discussion in *MMC* 126; Televantos (n73) ch 2.

79 J Hardman, ‘Sevilleja v Marex Financial Limited: Reflective Loss and the Autonomy of Company Law’ (2022) 85 MLR 232.

80 Not all business legal persons are provided for by the state. For example, the Scottish partnership has personality that arises automatically (Partnership Act 1890, s 4(2)). It does not, though, allow for perpetual succession, thus does not run counter to this argument—see Macgregor (n 44).

81 *MMC* 216–21.

82 Hardman, ‘Fixing the Misalignment’ (n 30).

83 *MMC* 213–14.

84 Vinogradoff (n 58).

85 EW Orts, *Business Persons: A Legal Theory of the Firm* (OUP 2013) 21–7.

86 *MMC* 8.

87 For an overview, see SK Ripken, *Corporate Personhood* (CUP 2019). This has long been the case—see WM Geldart, ‘Legal Personality’ (1911) 27 LQR 90, 94.

88 *MMC* ch 13.

89 Companies Act 2006, s 1(1).

90 It would be a ‘mistake’ to ‘confuse’ the two—see H Demsetz, ‘The Structure of Ownership and the Theory of the Firm’ (1983) 26 Journal of Law and Economics 375, 377. See also R Coase, ‘The Nature of the Firm’ (1937) 4 Economica 386, which set up the firm as the alternative organisational method to a market.

91 GM Hodgson, ‘Taxonomic Definitions in Social Science, with Firms, Markets and Institutions as Case Studies’ (2019) 15 Journal of Institutional Economics 207.

92 See J Hardman, ‘The Nexus of Contracts Revisited: Delineating the Business, the Firm, and the Legal Entity’ (2022) 34 Bond LR 1.

93 eg partnership, which has its own conceptual approach in respect of personality—see H Wells, ‘The Personification of Partnership’ (2021) 74 Vand L Rev 1835.

94 Recent research indicates that companies tend to be very small—see J Hardman and G Ramírez Santos, ‘Empirical Evidence for the Continuing Need to “Think Small First” in UK Company Law’ (2023) 24 EBOR 117.

95 Hardman, ‘Looking Beyond Separate Legal Personality’ (n 32) 8.

96 A UK private company can be incorporated with one share of £0.00001, so legal capital rules in the UK do not mandate the presence of a corporate fund. See E Ferran, ‘Revisiting Legal Capital’ (2019) 20 EBOR 521.

97 S Deakin and others, ‘Legal Institutionalism: Capitalism and the Constitutive Role of Law’ (2017) 45 Journal of Comparative Economics 188, 196.

98 ibid 197. See discussion in Hardman, ‘Fixing the Misalignment’ (n 30) 11–12.

99 S Deakin and others, ‘What Is a Firm? A Reply to Jean-Philippe Robé’ (2021) 17 Journal of Institutional Economics 861.

100 *MMC* 215–16.

101 See Hardman, ‘Fixing the Misalignment’ (n 30) 12.

102 S Deakin and others, ‘A Further Reply to Jean-Philippe Robé’ (2022) 18 Journal of Institutional Economics 703, 703.

103 JP Robé, ‘The Legal Structure of the Firm’ (2011) 1 Accounting, Economics and Law: A Convivium art 5.

104 See AA Schwartz, ‘The Perpetual Corporation’ (2012) 80 Geo Wash L Rev 764.

105 *MMC* 65–9.

106 JP Robé, *Property, Power and Politics: Why We Need to Rethink the World Power System* (Bristol UP 2020) 11.

107 On which see Bratton (n 65) 410.

108 Jensen and Meckling (n 12) 310–11.

109 Hardman, ‘The Nexus of Contracts Revisited’ (n 92) 15.

110 There are a number of misconceptions by lawyers—see BR Cheffins, ‘What Jensen and Meckling Really Said About the Public Company’ in E Pollman and R Thompson (eds), *Research Handbook on Corporate Purpose and Personhood* (Elgar 2020).

111 Mayer (n 3) 149–52.

112 See PL Davies, ‘Shareholder Voice and Corporate Purpose: The Purposeless of Mandatory Corporate Purpose Statements’ in L Enriques and G Stampelli (eds), *Board–Shareholder Dialogue: Policy Debate, Legal Constraints and Best Practice* (CUP forthcoming); Hardman, ‘The Nexus of Contracts Revisited’ (n 92) 21–2, 31–3.

113 See GM Hodgson, ‘Much of the “Economics of Property Rights” Devalues Property and Legal Rights’ (2015) 11 Journal of Institutional Economics 683.

114 Campbell called this the ‘fish scale model’—see DT Campbell, ‘Ethnocentrism of Disciplines and the Fish-Scale Model of Omniscience’ in S Muzafer and WS Carolyn (eds), *Interdisciplinary Relationships in the Social Sciences* (Adeline 1969), as discussed in GM Hodgson, ‘Donald T Campbell on the Institutions of Scientific Knowledge and the Limits to Interdisciplinarity’ (2022) 18 Journal of Institutional Economics 969.

115 Compare D Ciepley, ‘Beyond Public and Private: Toward a Political Theory of the Corporation’ (2013) 107 American Political Science Review 139 with AA Singer, ‘The Corporation’s Governmental Provenance and Its Significance’ (2019) 35 Economics and Philosophy 283.

116 *MMC* ch 8.

117 eg to make the company be as efficient as possible for private actors—BR Cheffins, *Company Law: Theory, Structure and Operation* (reprint, OUP 2008) ch 6.

118 eg N Naffine, ‘Who Are Law’s Persons? From Cheshire Cats to Responsible Subjects’ (2003) 66 MLR 346.

119 eg see VAJ Kurki, ‘Can Nature Hold Rights? It’s Not as Easy as You Think’ (2022) 11 TEL 525.

120 N Lacey, ‘Philosophical Foundations of the Common Law: Social not Metaphysical’ in J Horder (ed), *Oxford Essays on Jurisprudence* (OUP 2000).

121 eg HJ Laski, ‘The Personality of Associations’ (1916) 29 Harv L Rev 404.

122 T Buocz and I Eisenberger, ‘Demystifying Legal Personhood for Non-Human Entities: A Kelsenian Approach’ (2023) 43 OJLS 32.

123 See *MMC* 199–207.

124 eg N Friedman, ‘Corporations as Moral Agents: Trade-Offs in Criminal Liability and Human Rights for Corporations’ (2020) 83 MLR 255.

125 eg A Grear, ‘Challenging Corporate “Humanity”: Legal Disembodiment, Embodiment and Human Rights’ (2007) 7 Human Rights Law Review 511.

126 ‘What We Talk About When We Talk About Persons: The Language of a Legal Fiction’ (2001) 114 Harv L Rev 1745.

127 *MMC* 221.

128 ibid.

129 eg Micheler (n 51).

130 *MMC* 264.

131 Those statutes which provided incorporation of companies if statutory procedures were followed—see Butler (n 71).

132 *MMC* 213.

133 Watson, ‘The Corporate Legal Person’ (n 29).

134 *MMC* 221.

135 *MMC* 218–21.

136 EM Dodd, ‘For Whom Are Corporate Managers Trustees?’ (1932) 45 Harv L Rev 1145, 1160.

137 Companies Act 2006, s 14.

138 Companies Act 2006, ss 15 and 16.

139 Economic Crime and Corporate Transparency Act 2023, ss 2–7, which will restrict incorporation.

140 *MMC* 278.

141 Especially the ‘corporate group’—see R Squire, ‘Strategic Liability in the Corporate Group’ (2011) 78 U Chi L Rev 605.

142 *MMC* 280.

143 eg *MMC* 221.

144 M Koessler, ‘The Person in Imagination or Persona Ficta of the Corporation’ (1949) 9 La L Rev 435.

145 *MMC* 264–5.

146 *MMC* 281. This analysis may not apply pre-1980s, when different approaches to regulation were often proposed. See discussion in Gindis, ‘On the Origins’ (n 12).

147 *MMC* ch 16.

148 For the UK, see A Keay and J Loughrey, ‘The Concept of Business Judgment’ (2019) 39 LS 36.

149 See CAE Goodhart and R Lastra, ‘Equity Finance: Matching Liability to Power’ (2021) 6 Journal of Financial Regulation 1.

150 One of the requirements is to hold a UK qualification of a type only conferred on natural persons—see Medical Act 1983, s 3. For a now outdated list of limitations, see MA Pickering, ‘The Company as a Separate Legal Entity’ (1968) 31 MLR 481.

151 Companies Act 2006, ss 58 and 59.

152 eg withdrawals can only be made under certain statutory circumstances (*Trevor v Whitworth* (1887) 12 App Cas 409), and then only from profit and not capital—see *Flitcroft’s Case* (1882) 21 Ch D 519, as discussed in *MMC* 145.

153 Corporation Tax Act 2010. This does not apply to partnerships—see discussion in A Televantos, ‘The Nature of Partnership Property and Equitable Interests’ (2023) 139 LQR 26.

154 M Bennett and RJG Claassen, ‘The Corporate Power Trilemma’ (2022) 84 Journal of Politics 2094.

155 For example, a proposal to introduce legal personality for English law partnerships was partially to mitigate practical problems caused by outsiders engaging with the partnership—Law Commission and Scottish Law Commission, *Partnership Law* (Law Com No 283, Scot Law Com No 192, 2003) paras 5.5–5.24.

156 R Tillman, ‘Apology and Ambiguity: Adolf Berle on Corporate Power’ (1974) 8 Journal of Economic Issues 111.

157 eg H Hansmann and R Kraakman, ‘Toward Unlimited Shareholder Liability for Corporate Torts’ (1991) 100 Yale LJ 1879.

158 D Ciepley, ‘Can Corporations Be Held to the Public Interest, or Even to the Law?’ (2019) 154 Journal of Business Ethics 1003.

159 Both jurisdictionally (R Romano, ‘Answering the Wrong Question: The Tenuous Case for Mandatory Corporate Laws’ (1989) 89 Colum L Rev 1599) and to alternative vehicles (LE Ribstein, ‘The Deregulation of Limited Liability and the Death of Partnership’ (1992) 70 Wash ULQ 417).

